# Association between Serum Total Testosterone Level and Bone Mineral Density in Middle-Aged Postmenopausal Women

**DOI:** 10.1155/2022/4228740

**Published:** 2022-08-17

**Authors:** JinXiao Yang, Guofei Kong, Xiaocong Yao, Zhongxin Zhu

**Affiliations:** ^1^Department of Urology, The First People's Hospital of Xiaoshan District, Xiaoshan Affiliated Hospital of Wenzhou Medical University, Hangzhou, Zhejiang 311200, China; ^2^Department of Osteoporosis Care and Control, The First People's Hospital of Xiaoshan District, Xiaoshan Affiliated Hospital of Wenzhou Medical University, Hangzhou, Zhejiang 311200, China; ^3^Department of Clinical Research, The First People's Hospital of Xiaoshan District, Xiaoshan Affiliated Hospital of Wenzhou Medical University, Hangzhou, Zhejiang 311200, China

## Abstract

**Background:**

Hormone status strongly affects women's health and quality of life. To date, studies investigating the association between total testosterone (T) level and bone mineral density (BMD) in women are limited and have yielded contradictory conclusions. The aim of our study was to examine the association between serum total T level and lumbar BMD in postmenopausal women aged 40–59 years.

**Methods:**

The study group included 1,058 women from the 2011–2016 National Health and Nutrition Examination Survey. Multiple regression analyses were used to evaluate the association between serum total T level and lumbar BMD.

**Results:**

After adjusting for covariates, there was a positive association between the serum total T level and lumbar BMD (*β*, 1.07; 95% confidence interval, 0.17–1.97). A non-linearity in this association was identified, with a point of inflection at 30 ng/dL.

**Conclusions:**

Serum total T level was positively associated with lumbar BMD in middle-aged postmenopausal women up to a T level >30 ng/dL. Therefore, increasing T level in women with a low serum total T level may have beneficial outcomes on bone health.

## 1. Background

Hormone status strongly affects women's health and quality of life, with age-associated estrogen deficiency and hormonal imbalance having been implicated in the pathogenesis of various diseases, including osteoporosis [1]. Postmenopausal osteoporosis is the most common type of osteoporosis and is characterized by low bone mineral density (BMD), which increases the risk of fractures [2, 3]. The cause of postmenopausal osteoporosis, however, remains to be clarified.

During a normal menstrual cycle, the ovaries produce estrogen, androgens, and progesterone [4]. While estrogen deficiency is generally considered to be associated with bone loss in postmenopausal women [5], age-related reduction in testosterone (T) levels might also seriously affect bone health via its action on the androgen receptor [6,7]. In aging men, hypogonadism is associated with reduced BMD, with androgen deprivation therapy being of potential benefit in this regard, as suggested by current guidelines [8]. Experimental data suggested that T influences bone directly via interactions with androgen receptors and indirectly via binding to estrogen receptor (ER) *α* and ER*β* after aromatization in adipose or different tissues [9]. However, studies investigating the association between serum total T levels and bone health among women is limited and have yielded contradictory conclusions [10–12]. Moreover, the rate of bone loss is notably high in the first few years of menopause for women [13]. Accordingly, our aim in this study was to evaluate the association between serum total T level and lumbar BMD in middle-aged postmenopausal women using data from the National Health and Nutrition Examination Survey (NHANES).

## 2. Materials and Methods

### 2.1. Data Source and Study Population

The NHANES is a large, ongoing, cross-sectional, population-level survey regarding the health and nutrition status of a nationally representative sample of the non-institutionalized population in the United States. The NHANES uses a complex, stratified, multistage probability sampling design. The survey protocols were approved by the Institutional Review Board of the National Center for Health Statistics, and all participants provided written consent for the use of their data for research.

**Figure 1:**
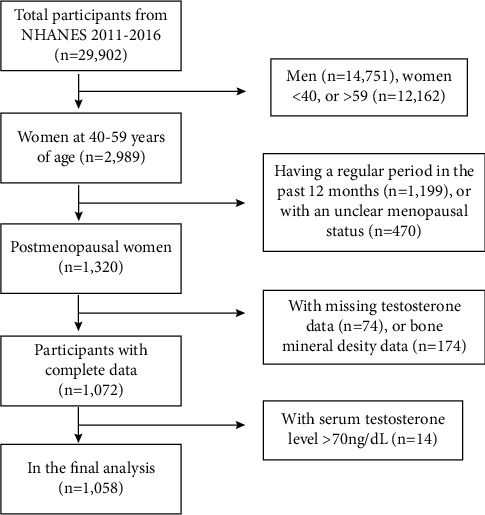
Flowchart of the selection of individuals from the 2011–2016 NHANES database.

For our study, we pooled data from the NHANES between 2011 and 2016. The study population was limited to postmenopausal women aged 40–59 years (*n* = 1,320). We excluded individuals with missing serum total T level (*n* = 74) or lumbar BMD (*n* = 174) data, as well as those with a serum total T level above the upper limit of normal (70 ng/dL; *n* = 14). After selection, 1,058 women were included in our final analysis (Figure 1).

### 2.2. Study Variables

The isotope dilution liquid chromatography tandem mass spectrometry method was used to measure the concentration of serum total T levels, based on the reference method of the National Institute for Standards and Technology [14]. Dual-energy X-ray absorptiometry was used to quantify lumbar BMD, acquired using a Hologic Discovery model A densitometer. The following covariates were included: age, race, body mass index (BMI), education level, income to poverty ratio, moderate activities, age since menopause, blood urea nitrogen, serum uric acid, total protein, serum phosphorus, and serum calcium. The detailed process for acquisition of these variables can be found on the NHANES website (https://www.cdc.gov/nchs/nhanes/).

### 2.3. Statistical Analyses

According to the analytical guideline edited by NCHS, data analyses took into account sampling weights. Weighted multiple regression analyses were then used to evaluate the association between serum total T level and lumbar BMD. Three models were constructed to provide statistical inference: model 1, no adjustment for covariates; model 2, adjustment for age and race; and model 3, adjustment for all covariates. We further performed smooth curve fitting to address potential non-linearity and performed a two-piecewise linear regression model when non-linearity was identified. Statistical analyses were performed using EmpowerStats and R software (version 3.4.3). Statistical significance was set at *P* < 0.05.

## 3. Results

Baseline characteristics for the 1,058 women included in our study group are presented in Table 1 by quartile of serum total T level. Compared to the Q4 group, women with lower serum total T levels had a higher level of blood urea nitrogen and lower BMI and lumbar BMD. As shown in Table 2, there was a positive association between serum total T level and lumbar BMD in all three regression models (model 1: *β* 1.65, 95% confidence interval (CI) 0.74–2.56; model 2: *β*, 1.43; 95% CI, 0.54–2.32; and model 3: *β*, 1.07; 95% CI, 0.17–1.97). *P* value for trend was significant for the three regression models across the quartile groups of serum T total levels. On subgroup analysis stratified by BMI (Table 3), the positive association remained significant for the 25–29.9 kg/m^2^ BMI group (*β*, 2.60; 95% CI, 0.73–4.47) but not for the <25 kg/m^2^ BMI group (*β*, 0.20; 95% CI, −1.81–2.21) or the ≥30 kg/m^2^ BMI group (*β*, 0.27; 95% CI, −0.93–1.47). However, the positive association was no longer significant after adjusting for covariates in subgroup analysis stratified by race (Table 3).

**Figure 2:**
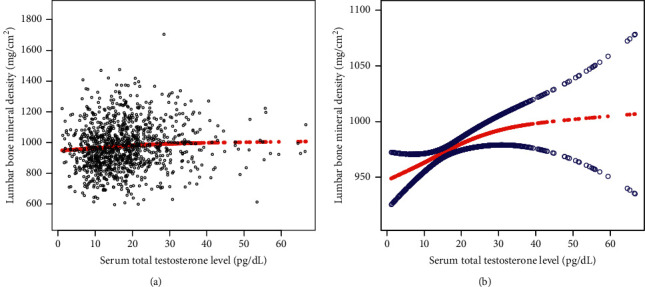
The association between serum total testosterone level and bone mineral density. (a) Each black point represents a sample. (b) Solid red line represents the smooth curve fit between variables. Blue bands represent the 95% of confidence interval from the fit (adjusted for age, race, body mass index, education level, income to poverty ratio, moderate activities, age since menopause, blood urea nitrogen, serum uric acid, total protein, serum phosphorus, and serum calcium).

The non-linear association between serum total T levels and lumbar BMD is shown in Figure 2, with the point of inflection point at 30 ng/dL (Table 4). For serum total T levels <30 ng/dL, the effect size of the serum total T level on BMD was 1.47 (95% CI, 0.19–2.75), with an effect size of 0.14 (95% CI, −2.15–2.44) for serum total T levels >30 ng/dL.

## 4. Discussion

The key finding of our study is the overall positive relationship between serum total T level and lumbar BMD in middle-aged postmenopausal women, with this association being linear up to a level of 30 ng/dL. Therefore, increasing testosterone in women with low T levels (<30 ng/dL) may improve bone health and, thus, outcomes of postmenopausal osteoporosis.

Bone growth and maintenance are significantly influenced by testosterone, which exerts strong androgenic and anabolic effects in both men and women [9]. Based on current evidence, it is unclear whether low serum T level is a potential risk factor for osteoporosis in men. Specifically, while some studies have supported a positive association between T levels and BMD [15–17], other have not [18–20]. Similarly, the association between serum T levels and BMD in women remains controversial. In a cross-sectional study of 64 postmenopausal women, no significant association was identified between serum T levels and BMD [10]. Nevertheless, a prior study did report a positive association between T concentrations and increased BMD in women [11]. Moreover, in women with classic congenital adrenal hyperplasia, the androgen excess provides a protective effect on BMD [12].

Our results revealed that higher serum total T level was significantly associated with higher lumbar BMD, up to a level of >30 ng/dL, with the positive association not retained after this point. A previous study has shown that high serum T levels in women are associated with adverse health effects, including type 2 diabetes, polycystic ovary syndrome, and breast and endometrial cancers [21]. Therefore, the balance between the potential benefits and risks of higher serum T levels needs to be comprehensively considered.

Testosterone plays a role in bone formation through its direct action on osteoblasts, via the androgen receptor, as well as has indirect effects on bone metabolism through its effect on various growth factors and cytokines [22]. Moreover, testosterone can promote osteoblast differentiation and apoptosis by increasing the expression level of androgen receptor [9, 23]. Moreover, T can be converted to estradiol by the aromatase enzyme, and estradiol binds to the estrogen receptor and exerts estrogenic action [24]. Further studies are needed to explore the effects of different T concentrations on bone metabolism.

A strength of our study is our use of a representative sample of postmenopausal women from the general population of the United States and the large sample size. The limitations of our study need to be acknowledged. First, due to the cross-sectional nature of the NHANES dataset used, a causal relationship between serum total T level and lumbar BMD could not be established. Second, data regarding additional potential confounders were unavailable in the database, such as levels of gonadotropin-releasing hormone, luteinizing hormone, follicle-stimulating hormone, and sex hormone-binding globulin (2011–2012 cycle). Our findings will need to be further validated including these additional confounders. Lastly, we excluded women with serum total T level >70 ng/dL; as such, our findings cannot be applied to these women.

## 5. Conclusion

Our population-based analysis of postmenopausal women revealed a positive association between serum total T level and lumbar BMD up to a serum total T level >30 ng/dL. There may therefore be a benefit of appropriately increasing testosterone in women with low serum total T levels (<30 ng/dL) on bone health.

## Figures and Tables

**Table 1:** Weighted characteristics of study population based on serum total testosterone level quartiles.

Serum total testosterone levels (ng/dL)	Q1 (≤11)	Q2 (>11, ≤16.15)	Q3 (>16.15, ≤21.55)	Q4 (>21.55)	*p* value
Age (years)	53.2 ± 4.8	52.2 ± 5.1	52.9 ± 4.7	52.6 ± 4.6	0.101
Race/ethnicity (%)					0.012
Non-Hispanic white	66.0	63.9	73.6	78.1	
Non-Hispanic black	12.9	13.2	11.3	11.1	
Mexican American	8.9	9.4	5.3	3.7	
Other race/ethnicity	12.2	13.4	9.7	7.0	
Education level (%)					0.024
Less than high school	11.5	16.5	11.2	13.5	
High school	20.4	20.6	20.1	29.3	
More than high school	68.2	62.9	68.6	57.2	
Age since menopause (years)	10.5 ± 7.9	8.3 ± 6.5	7.6 ± 5.9	8.1 ± 7.5	<0.001
Body mass index (kg/m^2^)	29.1 ± 7.0	30.5 ± 7.0	30.2 ± 6.8	31.6 ± 8.3	0.001
Income to poverty ratio	3.3 ± 1.6	3.0 ± 1.6	3.2 ± 1.7	3.2 ± 1.6	0.448
Moderate recreational activities (%)					0.525
Yes	44.7	44.0	49.3	43.6	
No	55.3	56.0	50.7	56.4	
Blood urea nitrogen (mmol/L)	5.1 ± 1.5	4.7 ± 1.6	4.8 ± 1.7	4.6 ± 1.8	0.004
Serum uric acid (umol/L)	288.0 ± 75.9	284.7 ± 65.7	296.0 ± 75.7	294.0 ± 72.3	0.259
Total protein (g/L)	70.4 ± 4.4	71.0 ± 4.8	70.2 ± 4.6	70.4 ± 4.5	0.284
Serum phosphorus (mmol/L)	1.26 ± 0.16	1.23 ± 0.16	1.26 ± 0.16	1.23 ± 0.15	<0.001
Serum calcium (mmol/L)	2.37 ± 0.09	2.35 ± 0.08	2.35 ± 0.08	2.36 ± 0.10	0.114
Lumbar bone mineral density (mg/cm^2^)	951.3 ± 139.5	986.1 ± 151.8	992.5 ± 149.0	1009.6 ± 147.6	<0.001

Mean ± SD for continuous variables: *p* value was calculated by weighted linear regression model. % for categorical variables: *p* value was calculated by the weighted chi-square test.

**Table 2:** Association between serum total testosterone levels (ng/dL) and lumbar bone mineral density (mg/cm^2^).

	Model 1 *β* (95% CI)	Model 2 *β* (95% CI)	Model 3 *β* (95% CI)
Serum total testosterone levels	1.65 (0.74, 2.56)^*∗∗∗*^	1.43 (0.54, 2.32)^*∗∗*^	1.07 (0.17, 1.97)^*∗*^
Serum total testosterone levels (quartile)			

Q1 (≤11)	Reference	Reference	Reference
Q2 (>11, ≤16.15)	34.79 (8.80, 60.79)	31.77 (6.36, 57.17)	29.27 (4.04, 54.50)
Q3 (>16.15, ≤21.55)	41.19 (15.90, 66.48)	36.83 (12.12, 61.55)	30.10 (5.40, 54.80)
Q4 (>21.55)	58.30 (33.43, 83.17)	50.31 (25.90, 74.71)	40.91 (16.26, 65.57)
P for trend	<0.001	<0.001	0.002

Model 1: no covariates were adjusted. Model 2: age and race were adjusted. Model 3: age, race, body mass index, education level, income to poverty ratio, moderate activities, age since menopause, blood urea nitrogen, serum uric acid, total protein, serum phosphorus, and serum calcium were adjusted. ^*∗*^*P* < 0.05, ^*∗∗*^*P* < 0.01, and ^*∗∗∗*^*P* < 0.001.

**Table 3:** Association between serum total testosterone levels (ng/dL) and lumbar bone mineral density (mg/cm^2^), stratified by body mass index (BMI) and race.

	Model 1 *β* (95% CI)	Model 2 *β* (95% CI)	Model 3 *β* (95% CI)
*Stratified by BMI*			
BMI (<25 kg/m^2^)	1.48 (−0.63, 3.60)	0.10 (−1.89, 2.10)	0.20 (−1.81, 2.21)
BMI (25–29.9 kg/m^2^)	2.37 (0.55, 4.20)^*∗*^	2.33 (0.53, 4.12)^*∗*^	2.60 (0.73, 4.47)^*∗∗*^
BMI (≥30 kg/m^2^)	1.01 (−0.19, 2.20)	0.81 (−0.37, 1.99)	0.27 (−0.93, 1.47)

*Stratified by race*			
Non-Hispanic white	1.53 (0.11, 2.95)^*∗*^	1.51 (0.09, 2.93)^*∗*^	1.09 (−0.36, 2.54)
Non-Hispanic black	2.09 (−0.02, 4.21)	2.02 (−0.06, 4.10)	1.81 (−0.29, 3.91)
Mexican American	−0.68 (−3.38, 2.03)	−1.04 (−3.63, 1.56)	−0.13 (−2.92, 2.67)
Other race/ethnicity	1.29 (−0.65, 3.23)	1.37 (−0.52, 3.26)	1.13 (−0.79, 3.06)

Model 1: no covariates were adjusted. Model 2: age and race were adjusted. Model 3: age, race, body mass index, education level, income to poverty ratio, moderate activities,, age since menopause, blood urea nitrogen, serum uric acid, total protein, serum phosphorus, and serum calcium were adjusted. ^*∗*^*P* < 0.05, ^*∗∗*^*P* < 0.01, and ^*∗∗∗*^*P* < 0.001.

**Table 4:** Threshold effect analysis of serum total testosterone level (ng/dL) on lumbar bone mineral density (mg/cm^2^) using two-piecewise linear regression model.

Lumbar bone mineral density	Adjusted *β* (95% CI), *p* value
Serum total testosterone level	
Fitting by standard linear model	1.07 (0.17, 1.97) 0.020
Fitting by two-piecewise linear model	
Inflection point	30 (ng/dL)
Serum total testosterone level <30 (ng/dL)	1.47 (0.19, 2.75) 0.025
Serum total testosterone level >30 (ng/dL)	0.14 (−2.15, 2.44) 0.903
Log likelihood ratio	0.386

Age, race, body mass index, education level, income to poverty ratio, moderate activities, age since menopause, blood urea nitrogen, serum uric acid, total protein, serum phosphorus, and serum calcium were adjusted.

## Data Availability

The data of this study are publicly available on the NHANES website.
